# Prevalence of Burnout and Associated Work-Related Factors Among Intensive Care Unit Nurses at Tertiary Healthcare Setting, Riyadh, Saudi Arabia

**DOI:** 10.3390/ijerph23060757

**Published:** 2026-06-04

**Authors:** Bridget Ndlovu, Bernard Hope Taderera

**Affiliations:** Department of Environmental Health, Faculty of Health Sciences, University of Johannesburg, Johannesburg 2028, South Africa; bridgetncedondlovu@gmail.com

**Keywords:** burnout, ICU nurses, emotional exhaustion, Maslach Burnout Inventory (MBI-HSS), nurse-to-patient ratio, shift work, occupational stress, mental health support, patient safety

## Abstract

**Highlights:**

**Public health relevance—How does this work relate to a public health issue?**

- Burnout among ICU nurses is a widespread occupational health issue that affects mental health, workforce sustainability, and patient safety.
- Understanding factors contributing to burnout, including workload, shift patterns, and social support, is essential for addressing occupational stress in high-intensity healthcare settings.

**Public health significance—Why is this work of significance to public health?**

- The study identifies structural and psychosocial determinants of burnout, providing evidence to help improve working conditions and prevent mental health deterioration among critical care staff.
- Findings underscore the importance of health system leadership, mental health support, and organisational policies in promoting well-being among healthcare professionals.

**Public health implications—What are the key implications or messages for practitioners, policy makers and/or researchers in public health?**

- Multi-level interventions, including staffing optimisation, mental health education, anonymous support, and leadership engagement, can mitigate burnout and enhance patient care quality.
- Results inform policy development for occupational health, highlighting the need for proactive strategies to safeguard nurse well-being to help sustain healthcare systems in pursuing universal health coverage.

**Abstract:**

Burnout among intensive care unit (ICU) nurses is an escalating occupational health concern due to the high psychological and physical demands of critical care, with implications for staff well-being, patient safety, and healthcare quality. Despite its importance, limited evidence exists on burnout among ICU nurses in Saudi Arabian tertiary hospitals. This study investigated the prevalence of burnout, associated factors, and potential interventions to reduce stigma and support mental health among ICU nurses at a tertiary healthcare setting, Saudi Arabia. A quantitative cross-sectional design was employed using the Maslach Burnout Inventory–Human Services Survey (MBI-HSS). Simple random sampling selected 250 registered ICU nurses with at least six months of experience. Data was analysed using SPSS v30 with descriptive statistics, and chi-square tests at a significance level of *p* < 0.05. Findings indicated a high prevalence of burnout, with 52% of nurses reporting elevated emotional exhaustion. Burnout was significantly associated with overtime hours (χ^2^ = 29.155, df = 12, *p* = 0.015), nurse-to-patient ratios (χ^2^ = 36.170, df = 20, *p* = 0.015), shift patterns (day: χ^2^ = 4.931, df = 8, *p* = 0.765; night: χ^2^ = 263 4.226, df = 8, *p* = 0.836; rotating: χ^2^ = 3.739, df = 4, *p* = 0.442), living arrangements ((χ^2^ = 13.153, df = 16, *p* = 0.662), and perceived impact on patient outcomes. Participants identified mental health education, anonymous support programmes, psychological check-ins, and leadership encouragement as helpful coping strategies. The study concludes that burnout among ICU nurses is influenced by workload, work schedules, and organisational support, underscoring the need for systemic interventions to enhance nurse well-being and sustain healthcare quality.

## 1. Introduction

Burnout among healthcare professionals is increasingly recognised as a significant public health concern with implications for workforce sustainability, patient safety, and health system performance. Intensive care unit (ICU) nurses are particularly vulnerable due to the high-acuity nature of critical care, frequent exposure to mortality, ethical dilemmas, and sustained emotional labour. Burnout was conceptualised in this study according to the Maslach Burnout framework as a multidimensional occupational syndrome resulting from chronic workplace stress that has not been successfully managed. It comprises three distinct but interrelated dimensions: emotional exhaustion (feelings of emotional depletion and fatigue), depersonalisation (detached or cynical attitudes toward patients and work), and reduced personal accomplishment (feelings of reduced competence and achievement in one’s professional role) [1]. Nursing is widely regarded as a high-risk profession in which chronic occupational stress is intensified by heavy workloads, time pressure, and limited opportunities for recovery, especially when nurses are unable to provide adequate care to each patient [2]. Evidence indicates that burnout has wide-ranging consequences at the individual and organisational levels. Physically, burnout has been associated with adverse health outcomes such as cardiovascular, respiratory, and gastrointestinal disorders, chronic fatigue, and metabolic disease [3]. Psychologically, individuals experiencing burnout may report anxiety, depression, insomnia, and emotional distress [4]. Beyond its impact on individual well-being, burnout also affects healthcare organisations through increased absenteeism, reduced job satisfaction, and higher staff turnover, all of which may negatively influence healthcare quality and patient outcomes [2].

First conceptualised by Freudenberger in 1974 as a response to chronic workplace stress, burnout is most commonly measured using the Maslach Burnout Inventory (MBI), which assesses emotional exhaustion, depersonalisation, and reduced personal accomplishment [5,6]. International evidence demonstrates considerable variability in burnout prevalence among ICU nurses, ranging from 6% to over 70%, depending on contextual and organisational factors [7]. Contextually, ICU nurses work in highly diverse healthcare environments characterised by differences in staffing levels, workload intensity, patient acuity, organisational support, ethical climate, and exposure to end-of-life care and futile treatment situations. Studies indicate that periods of heightened healthcare pressure, such as the COVID-19 pandemic, substantially increased emotional strain and burnout levels among ICU nurses [8].

Methodologically, variability may arise from differences in study designs, nurse populations, healthcare settings, burnout measurement approaches, and the country where the research was carried out [2]. For example, Ho and Lin’s study on futile care and burnout among ICU nurses highlighted how workplace conditions and emotional demands within critical care environments can significantly influence burnout outcomes [7]. Studies from Saudi Arabia report similarly concerning patterns, with high levels of emotional exhaustion (54.3%), depersonalisation (46.4%), and reduced personal accomplishment (24.3%) among ICU staff [9]. Comparable findings from the United Kingdom and Belgium [7] reinforce the global relevance of this issue and the need for context-sensitive interventions.

Burnout is increasingly linked to patient safety outcomes, including increased medical errors and compromised quality of care, aligning it with WHO priorities for strengthening health systems [10]. In Saudi Arabia, unique contextual pressures such as rapid healthcare expansion, staffing shortages, cultural expectations, and the demands associated with the Hajj season may exacerbate occupational stress among ICU nurses [11,12]. The COVID-19 pandemic further intensified these stressors, placing ICU nurses under unprecedented psychological and organisational strain [13]. Despite these challenges, empirical evidence focusing specifically on burnout among ICU nurses in Saudi Arabian tertiary hospitals remains limited and fragmented. For instance, Alharbi et al. [11] focused on compassion fatigue and its relationship with nurse-sensitive indicators rather than providing a detailed analysis of burnout dimensions or their occupational determinants within ICU settings. Similarly, Rugaan et al. [12] examined burnout among ICU healthcare workers during the Hajj season but were restricted to two tertiary hospitals, thereby limiting generalisability, and did not comprehensively assess key organisational factors such as nurse-to-patient ratios, overtime, or perceived impacts on patient outcomes. Additionally, Almaghrabi and Alsharif [13] addressed occupational health risks among nurses more broadly, with a focus on physical conditions rather than psychological outcomes such as burnout. Consequently, there remains a lack of comprehensive, context-specific evidence examining the determinants of emotional exhaustion among ICU nurses in Saudi Arabian tertiary hospitals, particularly in relation to staffing dynamics and workload pressures.

Understanding burnout within this context is essential for developing targeted institutional strategies that protect nurses’ well-being and maintain healthcare quality. Therefore, this study investigates the prevalence of burnout and its associated factors among ICU nurses at a tertiary healthcare setting in Saudi Arabia. By generating context-specific evidence, the study contributes to the broader public health discourse on occupational stress in healthcare settings and informs organisational interventions aimed at supporting a resilient nursing workforce.

## 2. Materials and Methods

### 2.1. Study Design and Population

This study employed a quantitative cross-sectional descriptive design to examine the prevalence and correlates of burnout among intensive care unit (ICU) nurses at a single point in time. This design was appropriate for identifying patterns and associations between burnout dimensions and work-related factors such as workload, shift patterns, and organisational support without manipulating study variables. Cross-sectional approaches are widely used in occupational health and nursing research due to their efficiency, cost-effectiveness, and ability to provide statistically robust estimates of health-related phenomena in large populations [14]. The design aligned with the study’s objective of describing the current state of burnout rather than determining causal relationships over time.

The study was conducted at a tertiary healthcare setting in Saudi Arabia, a leading tertiary healthcare institution established in 2004. The tertiary healthcare setting comprises four specialised hospitals with over 1200 beds and provides advanced medical, surgical, paediatric, and neonatal intensive care services. As a national referral centre for complex cases, the hospital places substantial clinical and emotional demands on critical care staff, offering a relevant context for examining burnout in high-intensity healthcare environments. The multicultural composition of the nursing workforce, including both Saudi nationals and expatriates, further enriched the study context by highlighting the interaction between cultural diversity, workload pressures, and occupational stress.

The target population included all registered nurses working in the Medical, Surgical, Paediatric, and Neonatal ICUs at a tertiary healthcare setting. These nurses are directly responsible for the care of critically ill patients and are therefore particularly susceptible to emotional exhaustion, depersonalisation, and reduced personal accomplishment, as conceptualised in Maslach’s Burnout Theory [15].

### 2.2. Sampling

A simple random sampling technique was employed to ensure that each eligible ICU nurse had an equal probability of selection, thereby reducing selection bias and improving representativeness [16]. The hospital’s human resources department provided a comprehensive list of all ICU nurses, which served as the sampling frame. Eligible participants were assigned unique identification numbers, and a random number generator was used to select the required sample. No statistical software was used for randomisation. Instead, a manual systematic selection approach was employed, whereby every fifth name on the compiled ICU nurse list was selected. This process was repeated iteratively until the required sample size was reached. This method ensured an element of randomness in participant selection while maintaining feasibility within the available administrative records.

The sample size was calculated using Cochran’s formula for large populations:

Where

- nn = sample size.
- ZZ = Z-value at 95% confidence level (1.96).
- pp = estimated proportion (0.5, to maximise sample size).
- ee = margin of error (0.05).

Adjusting for a finite population of 600 ICU nurses:

$$n=\frac{384.16}{1+\frac{384.16-1}{600}}=235n=\frac{384.16}{1+\frac{384.16-1}{600}}=235$$

This yielded an initial sample size of 384. Adjusting for the finite population of approximately 600 ICU nurses at a tertiary healthcare setting resulted in a final sample size of 235 participants. This ensured adequate statistical power to detect meaningful associations between burnout and its predictors. To account for the possibility of incomplete questionnaires, non-response, or participant withdrawal, the researchers recruited 250 participants, exceeding the minimum required sample size. However, all distributed questionnaires were completed and returned, resulting in a 100% response rate. Consequently, the final analysed sample comprised 250 participants, which further strengthened the statistical power and reliability of the study findings.

### 2.3. Eligibility Criteria

To be eligible, nurses had to be registered with the Saudi Commission for Health Specialties (SCFHS), employed full-time in an ICU at a tertiary healthcare setting, and have a minimum of six months of ICU work experience.

### 2.4. Data Collection

Nurses at a tertiary healthcare setting were invited to complete a structured, self-administered paper-based questionnaire (Supplementary Materials, File S1) designed to explore socio-demographic factors, work-related variables, burnout dimensions, and coping strategies. The questionnaire was divided into five sections: Section A—socio-demographics; Section B—work experience; Section C—occupational factors, including workload and shift patterns; Section D—burnout assessment using the Maslach Burnout Inventory–Human Services Survey (MBI-HSS); and Section E—access to resources, job satisfaction, and coping mechanisms. One open-ended question allowed participants to provide qualitative insights into coping strategies, while the remaining items were closed-ended.

Potential participants were provided with information sheets and consent forms and were briefed on voluntary participation, confidentiality, and the study’s purpose. Questionnaires were distributed in sealed envelopes to ICU nurses, who completed them at their convenience, typically requiring 20–30 min. Completed questionnaires were returned anonymously to collection boxes to ensure privacy and encourage candidates’ responses. Data collection occurred between August and September 2025, with reminders provided during the study period to optimise participation. Participants were allowed a single attempt to complete the questionnaire, and no time limit was imposed, allowing them to respond at their convenience without pressure.

### 2.5. Reliability and Validity of the Instrument

#### 2.5.1. Reliability

The reliability of the questionnaire was evaluated through a pilot study involving ten ICU nurses who were not included in the main study sample. Feedback from the pilot helped refine the clarity and wording of the instrument. Internal consistency was evaluated using Cronbach’s alpha, with coefficients of ≥0.70 considered acceptable.

#### 2.5.2. Validity

Content validity was established by mapping each questionnaire item against Maslach’s Burnout Theory. Items from the Maslach Burnout Inventory–Human Services Survey (MBI-HSS) maintained their validated structure, while newly developed items on occupational and organisational factors were reviewed and refined through expert consensus. The expert panel comprised five professionals selected based on predefined criteria, including clinical experience in intensive care nursing, academic qualifications in nursing or related health sciences, and expertise in research methodology and instrument development. The experts independently reviewed the instrument for relevance, clarity, and representativeness of the constructs under investigation, specifically burnout and its associated factors.

Consensus was achieved through a structured review process in which experts provided individual ratings and qualitative feedback on each item. Revisions were made based on areas of agreement and recurring suggestions, and items were refined until satisfactory agreement was reached among the panel. This process ensured comprehensive coverage of burnout dimensions, work-related stressors, coping resources, and job satisfaction, enhancing both the theoretical alignment and measurement accuracy of the instrument.

### 2.6. Data Analysis

Quantitative data collected through the structured questionnaire were coded and entered into the Statistical Package for the Social Sciences (SPSS) Version 30. Prior to analysis, the dataset was screened for completeness, consistency, and outliers to ensure reliability and validity. The screening process involved checking for missing responses, inconsistent entries, and extreme values that could potentially distort the findings. To minimise the risk of bias and enhance the credibility of the data preparation process, the screening was conducted independently by more than one reviewer, with discrepancies discussed and resolved collaboratively. This multiple-reviewer approach strengthened the reliability and validity of the dataset prior to statistical analysis. Descriptive statistics, including frequencies, percentages, means, and standard deviations, were computed to summarise the demographic characteristics, work-related factors, burnout dimensions, and coping strategies of ICU nurses. Inferential analyses were performed to examine associations between key variables. Chi-square tests were used to assess relationships between categorical variables such as demographic factors, work schedules, nurse-to-patient ratios, overtime, and burnout indicators (emotional exhaustion and depersonalisation). Statistical significance was set at *p* ≤ 0.05. Variables demonstrating significant associations were further examined to identify potential risk factors contributing to burnout among nurses.

### 2.7. Ethics Approval

This study was conducted in accordance with the Declaration of Helsinki and approved by the University of Johannesburg Faculty Academic Research Ethics Committee (REC-3527-2025, 20 May 2025) and a tertiary healthcare setting, Institutional Review Board (IRB-25-317, 22 June 2025). In addition, permission was obtained from the hospital management to access the nursing staff lists.

## 3. Results

### 3.1. Demographic Profile

Overall, 250 ICU nurses consented to participate in the study, and all completed the survey, resulting in a 100% response rate (Table 1). The mean age of participants was 34.66 years (SD = 8.8), with the majority (40.8%, *n* = 102) aged 30–39 years, reflecting a relatively young workforce. Most respondents were female (68.4%, *n* = 171). Marital status was almost evenly split, with 46.8% (*n* = 117) single and 46.0% (*n* = 115) married, while smaller proportions were widowed (2.8%, *n* = 7) or divorced (4.4%, *n* = 11). Regarding living arrangements, over half of the participants (51.6%, *n* = 129) lived alone, 36.0% (*n* = 90) lived with family, and 7.2% (*n* = 18) lived with their children. Frequency of family contact was generally low, with 33.6% (*n* = 84) seeing family annually, 20.0% (*n* = 50) every six months, 11.2% (*n* = 28) every three months, and 35.2% (*n* = 88) in the “other” category. Most respondents described their support system as moderate (57.6%, *n* = 144), followed by weak (34.4%, *n* = 86) and strong (8.0%, *n* = 20). The majority held a bachelor’s degree (60.8%, *n* = 152), followed by a diploma (23.6%, *n* = 59), master’s degree (13.2%, *n* = 33), and doctorate (2.4%, *n* = 6). Slightly less than half of the participants (46.4%, *n* = 116) reported having an ICU-specific qualification, while the remaining 53.6% (*n* = 134) did not.

### 3.2. Workload, Shift Characteristics, and Staffing Patterns

The results in Table 2 illustrate workload, shift patterns, and nurse-to-patient ratios among ICU nurses (*n* = 250). Most participants (88.4%, *n* = 221) reported working 48 h per week, with the majority (99.6%, *n* = 249) working 12 h shifts. Rotating shifts were the most common, with 82.4% (*n* = 206) of nurses reporting participation, whereas day shifts were less frequent (17.2%, *n* = 43). Night shifts were reported by 19.6% (*n* = 49) of respondents. Regarding overtime, 60% (*n* = 150) reported working an additional 12 h weekly, and 74.4% (*n* = 186) indicated that overtime occurred occasionally. Despite this workload, only 36.0% (*n* = 90) expressed satisfaction with their current shift schedule, while 64.0% (*n* = 160) were dissatisfied. Nurse-to-patient ratios predominantly ranged from 1:1 to 1:3, with 52.0% (*n* = 130) reporting a 1:2 ratio. Most nurses (75.6%, *n* = 189) indicated that the nurse-to-patient ratio occasionally exceeded standard recommendations, although 69.2% (*n* = 173) perceived this had a neutral effect on patient outcomes. Staffing adequacy was limited, with 50.0% (*n* = 125) indicating that staffing sometimes met patient needs and 39.6% (*n* = 99) reporting it rarely did. High workloads contributed to professional challenges: 59.2% (*n* = 148) reported increased workload, 54.0% (*n* = 135) experienced reduced time for patient care, 58.8% (*n* = 147) reported increased stress, and 39.6% (*n* = 99) perceived a higher risk of errors. These findings suggest that ICU nurses face demanding work conditions, with frequent overtime, rotating shifts, and high nurse-to-patient ratios contributing to stress and potential impacts on patient care.

### 3.3. Burnout Assessment

As shown in Table 3, the majority of ICU nurses reported taking breaks infrequently during their shifts, with 58.8% (*n* = 147) indicating they rarely took breaks and 19.2% (*n* = 48) reporting never taking breaks. Emotional exhaustion was common, with 49.6% (*n* = 124) of participants reporting they sometimes felt emotionally drained after their shifts, while 3.6% (*n* = 9) reported often or always feeling drained. Regarding depersonalisation, 50.8% (*n* = 127) of nurses sometimes felt detached or indifferent toward their patients, and 18.0% (*n* = 45) reported often or always experiencing these feelings. Physical symptoms related to work stress, such as headaches and fatigue, were reported sometimes by 34.8% (*n* = 87) of participants, with 7.6% (*n* = 19) reporting often or always experiencing such symptoms. Perceived colleague support during stressful situations was moderate, with 32.0% (*n* = 80) reporting sometimes, while 62.8% (*n* = 157) reported rarely or never receiving support. Furthermore, only 29.2% (*n* = 73) of nurses had sought formal support, such as counselling or peer support, for stress or trauma, whereas 70.8% (*n* = 177) had not.

### 3.4. Personal Accomplishment Assessment

As shown in Table 4, the findings relating to the Personal Accomplishment subscale revealed generally moderate perceptions of professional efficacy and achievement among ICU nurses. Regarding whether respondents felt a sense of personal accomplishment from their work (Q41), the majority of respondents reported sometimes experiencing personal accomplishment (63.2%, *n* = 158). Smaller proportions indicated that they rarely (23.6%, *n* = 59) or never (9.2%, *n* = 23) experienced such feelings, while very few respondents reported experiencing personal accomplishment often (3.6%, *n* = 9) or always (0.4%, *n* = 1). The item yielded a mean score of M = 2.62 (SD = 0.719), suggesting moderate but inconsistent feelings of professional achievement among the nurses.

Similarly, in relation to whether respondents felt they were making an impact on patients’ lives (Q42), just over half of the respondents indicated sometimes (51.6%, *n* = 129), while 26.4% (*n* = 66) reported rarely and 19.6% (*n* = 49) reported never. Only a very small proportion indicated often (1.6%, *n* = 4) or always (0.8%, *n* = 2). The mean score for this variable was M = 2.38 (SD = 0.842), indicating relatively low perceptions of impact and professional effectiveness.

### 3.5. Occupationa Related Factors

The relationships between ICU nurses’ demographic and occupational variables and the frequency of feeling emotionally drained after shifts are presented in Table 5. Overall, no statistically significant associations were observed between emotional exhaustion and the number of weekly working hours (χ^2^ = 10.235, df = 16, *p* = 0.854), average daily shift length (χ^2^ = 1.754, df = 4, *p* = 0.781), or shift type (day: χ^2^ = 4.931, df = 8, *p* = 0.765; night: χ^2^ = 4.226, df = 8, *p* = 0.836; rotating: χ^2^ = 3.739, df = 4, *p* = 0.442). Similarly, living arrangements (χ^2^ = 13.153, df = 16, *p* = 0.662) and satisfaction with shift schedules (χ^2^ = 3.202, df = 4, *p* = 0.525) were not significantly associated with emotional exhaustion. In contrast, several occupational factors demonstrated significant associations with emotional exhaustion. Nurses’ average overtime hours per week were significantly related to feeling emotionally drained (χ^2^ = 29.155, df = 12, *p* = 0.015), indicating that increased overtime contributed to higher levels of emotional exhaustion. Additionally, the nurse-to-patient ratio (χ^2^ = 36.170, df = 20, *p* = 0.015) and the frequency with which the ratio exceeded recommended standards (χ^2^ = 22.381, df = 12, *p* = 0.033) were significantly associated with emotional exhaustion, suggesting that heavier patient loads and staffing pressures exacerbate feelings of fatigue among ICU nurses. Perceived effects of nurse-to-patient ratios on patient outcomes were also significantly associated with emotional exhaustion (χ^2^ = 31.382, df = 8, *p* < 0.001). Regarding work-related challenges, reduced time for patient care (χ^2^ = 18.572, df = 8, *p* = 0.017) and increased stress due to staffing levels (χ^2^ = 9.554, df = 4, *p* = 0.049) were significantly associated with emotional exhaustion. However, increased workload (χ^2^ = 17.530, df = 12, *p* = 0.131), higher risk of errors (χ^2^ = 7.128, df = 4, *p* = 0.129), and other staffing-related challenges (χ^2^ = 1.065, df = 4, *p* = 0.900) were not significantly related.

The associations between ICU nurses’ demographic and occupational characteristics and feelings of detachment or indifference towards patients are presented in Table 6. A significant relationship was observed between weekly working hours and detachment (χ^2^ = 42.280, df = 16, *p* < 0.001), indicating that nurses with longer work hours reported higher levels of detachment. Similarly, living arrangements were significantly associated with detachment (χ^2^ = 38.594, df = 16, *p* = 0.001), as were combined responses on living arrangements across categories (χ^2^ = 62.178, df = 24, *p* < 0.001), suggesting that nurses with limited household support or living alone were more likely to experience emotional distancing from patients. Additional occupational factors also showed significant associations. Nurse-to-patient ratio in the ICU was significantly related to detachment (χ^2^ = 42.941, df = 20, *p* = 0.002), with higher patient loads corresponding to increased reports of indifference. Perceived adequacy of staffing levels similarly influenced detachment (χ^2^ = 38.575, df = 16, *p* = 0.001), highlighting that nurses who considered staffing insufficient were more likely to feel detached. Work-related challenges stemming from the current nurse-to-patient ratio were also significant predictors of detachment. Increased workload (χ^2^ = 65.538, df = 12, *p* < 0.001), reduced time for patient care (χ^2^ = 29.080, df = 8, *p* < 0.001), increased stress (χ^2^ = 14.730, df = 4, *p* = 0.005), and other staffing-related challenges (χ^2^ = 12.332, df = 4, *p* = 0.015) were all associated with higher levels of detachment. Conversely, other variables, including shift type (day: χ^2^ = 7.559, df = 8, *p* = 0.478; night: χ^2^ = 9.971, df = 8, *p* = 0.267; rotating: χ^2^ = 6.679, df = 4, *p* = 0.154), average daily shift length (χ^2^ = 2.434, df = 4, *p* = 0.656), overtime frequency (χ^2^ = 13.700, df = 12, *p* = 0.320), nurse-to-patient ratio exceeding recommendations (χ^2^ = 15.563, df = 12, *p* = 0.212), perceived effects on patient outcomes (χ^2^ = 10.314, df = 8, *p* = 0.244), and higher risk of errors (χ^2^ = 0.357, df = 4, *p* = 0.986) were not significantly associated with detachment.

### 3.6. Coping Strategies Against Burnout

Table 7 presents ICU nurses’ perceptions of measures that could reduce stigma associated with seeking help for burnout. The majority of respondents supported encouragement from leadership to seek help (69.2%, *n* = 173) as a key strategy, highlighting the influential role of managerial support in fostering a psychologically safe work environment. Anonymous support programmes were also widely endorsed (61.2%, *n* = 153), suggesting that confidentiality and privacy are critical in promoting help-seeking behaviours among nurses. About half of the participants indicated that regular mental health check-ins (50.8%, *n* = 127) and education and training in mental health (46.8%, *n* = 117) could be beneficial. In contrast, very few respondents identified other measures (5.2%, *n* = 13), indicating that most perceived the proposed interventions as comprehensive.

## 4. Discussion

In this study, conducted among ICU nurses in a tertiary hospital setting, over half of the respondents (52%) reported elevated levels of emotional exhaustion, indicating a high prevalence of burnout. This finding is consistent with the Maslach Burnout Theory, which identifies emotional exhaustion as the first and most critical dimension of burnout, reflecting fatigue, depletion, and emotional overextension due to sustained occupational stress [17]. Emotional exhaustion in ICU settings is common, given the continuous exposure to critically ill patients, high patient acuity, and organisational pressures. Similar trends have been observed internationally. For example, Bruyneel et al. [18] reported a 68% burnout rate among ICU nurses in Belgium during the COVID-19 pandemic, while Ramirez-Elvira et al. [2] found a global prevalence of 44%. In Saudi Arabia, Awajeh, Issa, Rasheed and Amirah [5] and Shbeer and Ageel [9] reported rates of 65% and 62%, respectively. The slightly lower prevalence in the current study may reflect partial recovery from post-pandemic stressors or differences in institutional support structures. For example, studies conducted during the COVID-19 pandemic reported higher rates of emotional exhaustion [9,18], while those conducted before the pandemic generally reported comparatively lower rates [5].

Emotional exhaustion was significantly associated with living arrangements (χ^2^ = 163.711, df = 16, *p* < 0.001), highlighting the role of personal and social support in buffering or exacerbating burnout. Nurses living alone or away from family were more likely to report high exhaustion, consistent with Yanbei, Dongdong, Yun, Ning and Fengping [1], who observed that limited social interaction and poor work–life balance increase susceptibility to emotional depletion. Weekly overtime hours were also significantly associated with emotional exhaustion (χ^2^ = 29.155, df = 12, *p* = 0.015), confirming that prolonged work hours contribute to physiological and psychological strain [19,20]. In contrast, total weekly working hours (χ^2^ = 10.235, df = 16, *p* = 0.854) and average shift length (χ^2^ = 1.754, df = 4, *p* = 0.781) were not significant predictors, suggesting that intensity and emotional load, rather than total time worked, drive exhaustion [21,22]. Furthermore, the non-significant difference observed may suggest that it is not merely the duration or structure of working hours that contributes to burnout, but rather the intensity and unpredictability of workload, as reflected in overtime demands and staffing adequacy. These results highlight the complexity of burnout determinants and suggest that workload quality and staffing conditions may be more critical predictors than quantitative measures of working hours.

Nurse-to-patient ratios were also significantly associated with emotional exhaustion (χ^2^ = 36.170, df = 20, *p* = 0.015), confirming that workload intensity and staffing inadequacy are structural determinants of burnout. High patient loads increase stress, error rates, and emotional fatigue [23,24]. Nurses caring for more than two ICU patients simultaneously are three times more likely to experience emotional exhaustion [18]. Emotional exhaustion was further associated with perceived negative impacts on patient outcomes (χ^2^ = 31.382, df = 8, *p* < 0.001), supporting the idea that burnout can reduce engagement and empathy, leading to compromised care [25,26].

Depersonalisation, reflecting emotional detachment and cynicism toward patients, was significantly associated with weekly working hours (χ^2^ = 42.280, df = 16, *p* < 0.001), living arrangements (χ^2^ = 62.178, df = 24, *p* < 0.001), and nurse-to-patient ratios (χ^2^ = 42.941, df = 20, *p* = 0.002). This is clinically important, as depersonalisation represents a maladaptive coping response within the Maslach Burnout framework, where prolonged exposure to emotional exhaustion leads healthcare workers to psychologically distance themselves from patients as a protective mechanism [17]. From a patient safety perspective, depersonalisation is particularly concerning in intensive care settings because it can compromise therapeutic communication, reduce attentiveness to patient needs, and weaken the nurse–patient relationship, all of which are critical for high-quality critical care delivery [27].

High workload, limited patient care time (χ^2^ = 29.080, df = 8, *p* < 0.001), and irregular shifts were also associated with depersonalisation and physical fatigue [28,29]. This detachment may inadvertently increase the risk of clinical errors, reduce vigilance in monitoring critically ill patients, and negatively affect patient satisfaction and overall care outcomes. The significant association between depersonalisation and nurse-to-patient ratios is particularly noteworthy, as understaffing increases workload intensity and reduces opportunities for meaningful patient interaction, thereby heightening emotional strain and fostering detachment [30]. Similarly, living arrangements may reflect underlying social support structures, where limited external support could exacerbate emotional exhaustion and increase reliance on distancing behaviours as a coping strategy [31].

Furthermore, the finding that both early-career and long-tenure nurses exhibited higher levels of burnout-related characteristics is consistent with the existing literature [32,33], suggesting that insufficient coping mechanisms in newer staff and cumulative exposure to chronic stressors in experienced nurses may both contribute to depersonalisation. Clinically, this highlights the need for targeted interventions such as emotional resilience training, reflective practice sessions, adequate staffing levels, and supportive supervision to mitigate the progression of depersonalisation and protect patient safety in ICU environments.

Reduced personal accomplishment, reflecting diminished feelings of professional competence, achievement, and effectiveness in patient care, was evident among ICU nurses, with most respondents reporting that they only sometimes experienced a sense of accomplishment from their work (63.2%, *n* = 158) or felt they were making an impact on patients’ lives (51.6%, *n* = 129). Lower perceptions of professional efficacy were further reflected in the mean scores for personal accomplishment (M = 2.62, SD = 0.719) and perceived impact on patients’ lives (M = 2.38, SD = 0.842). These findings align with the Maslach Burnout Theory, which conceptualises reduced personal accomplishment as one of the three core dimensions of burnout arising from prolonged occupational stress and chronic emotional demands [17]. In ICU settings, repeated exposure to critically ill patients, high mortality rates, ethical dilemmas, and emotionally demanding care environments may progressively undermine nurses’ perceptions of competence and achievement [27]. Organisational stressors such as heavy workloads, staffing shortages, overtime demands, and limited opportunities for recovery and professional recognition have also been associated with lower levels of personal accomplishment among nurses [28,29]. Furthermore, exposure to futile care situations and limited positive patient outcomes may reduce nurses’ sense of professional impact and meaning, contributing to diminished work satisfaction and psychological well-being [34].

The study also explored coping strategies and interventions to reduce burnout and stigma. Nearly half of participants (46.8%) endorsed mental health education and training, over 60% supported anonymous support programmes, and 50.8% favoured regular mental health check-ins. The strongest endorsement (69.2%) was for leadership encouragement, underscoring the pivotal role of managerial support in promoting psychological safety and destigmatising help-seeking [32,35]. These findings are consistent with the Job Demands–Resources model, which suggests that both the reduction in job demands and the enhancement of resources, such as emotional support, are necessary to mitigate burnout [36].

Anonymous support programmes and regular check-ins were valued, reflecting ongoing stigma around mental health in clinical environments [37,38]. Leadership encouragement was particularly influential, with transformational and compassionate leadership reducing burnout and fostering open dialogue about mental health [39,40]. Integrated interventions combining organisational strategies (e.g., peer support, workload redistribution) and individual-level interventions (e.g., mindfulness, cognitive-behavioural approaches) are most effective [41,42,43].

Burnout among ICU nurses has significant public health implications. Persistent emotional exhaustion and depersonalisation compromise nurse well-being, patient safety, and healthcare system resilience [18]. Findings highlight the need for policy-level interventions, including improved staffing, regulated overtime, mental health surveillance, and stigma reduction programmes, which align with WHO recommendations on psychosocial well-being at work [10]. Targeted resilience-building interventions, peer support, and leadership development can reduce turnover, enhance coping capacity, and contribute to Sustainable Development Goal 3: Good Health and Well-being.

Limitations include cross-sectional design, self-reported data, single-site sampling, and the absence of qualitative insights, restricting causal inference and contextual depth due to a lack of funding. The reliance on self-reported measures introduces the potential for response and social desirability. Moreover, the single-site setting may restrict the generalisability of the findings. Furthermore, the absence of qualitative data limits in-depth understanding of contextual and experiential factors. Future studies will need to carry out a case study focusing on all healthcare facilities of different types and at different levels, using qualitative and quantitative methods in a funded research project over a period of time for the generation of a conceptual framework that can be used for a more in-depth understanding of the prevalence of burnout and associated work-related factors within the healthcare system of Saudi Arabia. Nevertheless, strengths include the use of a validated MBI-HSS tool, a robust sample size (*n* = 250), and systematic analysis using SPSS. The focus on ICU nurses and exploration of coping strategies provides applied relevance to hospital administrators, policymakers, and mental health practitioners.

To strengthen the policy relevance of the study within the context of Saudi Vision 2030 healthcare reforms, it is recommended that Saudi health regulators and hospital managers prioritise the implementation of targeted workforce well-being strategies within intensive care units. These should include the establishment of mandatory nurse-to-patient staffing standards, routine monitoring of burnout indicators using validated tools such as the MBI-HSS, and the integration of structured psychological support programmes for ICU nurses. In alignment with Vision 2030’s focus on improving healthcare quality, patient safety, and workforce sustainability, hospital management should also invest in resilience-building initiatives, continuous professional development, and leadership training aimed at early identification and mitigation of burnout risk. Furthermore, organisational policies should address workload distribution, reduce excessive overtime, and strengthen supportive supervision structures to enhance nurse retention and improve patient care outcomes.

## 5. Conclusions

This study provides insights into prevalence, contributing factors, and coping strategies for burnout among ICU nurses, including emotional exhaustion, depersonalisation, workload intensity, overtime, living arrangements, and nurse-to-patient ratios. Nurses identified mental health education, anonymous support programmes, regular check-ins, and leadership encouragement as key strategies to reduce burnout and stigma. The findings highlight the importance of both individual-level and organisational-level interventions to support nurse well-being and sustain high-quality patient care. Further research is needed to explore longitudinal patterns of burnout, the effectiveness of targeted interventions, and the influence of organisational culture and social support on nurse mental health across different healthcare contexts and settings.

## Figures and Tables

**Table 1:** Participant characteristics (*n* = 250).

Variables	Frequency (*n*)	Percent (%)
Age		
18–29	82	32.8
30–39	102	40.8
40–49	45	18.0
50–60	21	8.4
Gender		
Male	79	31.6
Female	171	68.4
Marital Status		
Married	115	46.0
Single	117	46.8
Widowed	7	2.8
Divorced	11	4.4
Who do you currently live with		
My children	18	7.2
My family	90	36.0
Alone	129	51.6
Other	13	5.2
How often do you see your family?		
Every 3 months	28	11.2
Every 6 months	50	20.0
Annually	84	33.6
Others	88	35.2
How would you describe your current support system?		
Weak	86	34.4
Moderate	144	57.6
Strong	20	8.0
What is your highest educational level?		
Doctorate	6	2.4
Master’s Degree	33	13.2
Bachelor’s Degree	152	60.8
Diploma	59	23.6
Do you have any ICU qualifications?		
Yes	116	46.4
No	134	53.6

**Table 2:** Workload, shift patterns, and nurse-to-patient ratios among ICU nurses (*n* = 250).

Variables	Category	Frequency (*n*)	Percent (%)
Weekly working hours	12	2	0.8
	46	4	1.6
	48	221	88.4
	60	21	8.4
	72	2	0.8
Average shift length in hours	12	249	99.6
	13	1	0.4
Shift type	Day shift—Yes	43	17.2
	Day shift—No	207	82.8
	Night shift—Yes	49	19.6
	Night shift—No	201	80.4
	Rotating shift—Yes	206	82.4
	Rotating shift—No	44	17.6
Overtime hours per week	None	10	4.0
	12 h	150	60.0
	24 h	30	12.0
	3 h	2	0.8
	48 h	5	2.0
	8 h	2	0.8
	Others	51	20.4
Overtime frequency	Daily	1	0.4
	Weekly	52	20.8
	Occasionally	186	74.4
	Never	11	4.4
Satisfaction with current shift schedule	Yes	90	36.0
	No	160	64.0
Nurse-to-patient ratio	1:1	57	22.8
	1:2	130	52.0
	1:3	45	18.0
	1:4	2	0.8
	Others	16	6.4
Frequency ratio exceeds standard	Rarely	34	13.6
	Occasionally	189	75.6
	Frequently	24	9.6
	Always	3	1.2
Effect on patient outcomes	Negatively	24	9.6
	Neutral	173	69.2
	Positively	53	21.2
Adequacy of staffing	Never	11	4.4
	Rarely	99	39.6
	Sometimes	125	50.0
	Often	14	5.6
	Always	1	0.4
Challenges due to nurse-to-patient ratio	Increased workload	148	59.2
	Reduced time for patient care	135	54.0
	Increased stress	147	58.8
	Higher risk of errors	99	39.6
	Other	13	5.2

**Table 3:** Frequency and percentage of ICU nurses’ work-related stress and coping behaviours (*n* = 250).

Variables	Category	Frequency	Percent (%)
How often do you take breaks during your shift	Never	48	19.2
	Rarely	147	58.8
	Occasionally	53	21.2
	Regularly	2	0.8
How often do you feel emotionally drained after your shift	Never	26	10.4
	Rarely	91	36.4
	Sometimes	124	49.6
	Often	8	3.2
	Always	1	0.4
How often do you feel detached or indifferent towards your patients	Never	5	2.0
	Rarely	73	29.2
	Sometimes	127	50.8
	Often	39	15.6
	Always	6	2.4
Do you experience physical symptoms related to work stress	Never	36	14.4
	Rarely	108	43.2
	Sometimes	87	34.8
	Often	16	6.4
	Always	3	1.2
Do you feel supported by colleagues during stressful situations	Never	59	23.6
	Rarely	98	39.2
	Sometimes	80	32.0
	Often	11	4.4
	Always	2	0.8
Have you ever sought support for stress or trauma	Yes	73	29.2
	No	177	70.8

Note. *n* = 250 ICU nurses. Physical symptoms include headaches, fatigue, and other stress-related conditions.

**Table 4:** Personal accomplishment (*n* = 250).

Variables	Frequency (*n*)	Percent	Mean	Std. Deviation
Do you feel a sense of personal accomplishment from your work			2.62	0.719
Never	23	9.2		
Rarely	59	23.6		
Sometimes	158	63.2		
Often	9	3.6		
Always	1	0.4		
Do you feel that you are making an impact on patients’ lives			2.38	0.842
Never	49	19.6		
Rarely	66	26.4		
Sometimes	129	51.6		
Often	4	1.6		
Always	2	0.8		

**Table 5:** The frequency of emotional exhaustion among ICU nurses and its association with various work-related factors.

Variables	How Often Do You Feel Emotionally Drained After Your Shift?					Chi-Square (df)	*p*-Value
	Never	Rarely	Sometimes	Often	Always		
How many hours do you work per week						10.235 (16)	0.854
12	0.0%	1.1%	0.8%	0.0%	0.0%		
46	3.8%	1.1%	1.6%	12.5%	0.0%		
48	84.6%	90.1%	88.7%	87.5%	100.0%		
60	11.5%	7.7%	8.9%	0.0%	0.0%		
What is the average length (hours) per day shift						1.754 (4)	0.781
12	100.0%	100%	100.0%	100.0%	100.0%		
What types of shifts do you work							
Day shift						4.931 (8)	0.765
No	76.9%	84.6%	81.5%	100.0%	100.0%		
Day shift	23.1%	14.3%	18.5%	0.0%	0.0%		
Night shift						4.226 (8)	0.836
No	76.9%	80.2%	79.8%	100.0%	100.0%		
Night shift	23.1%	19.8%	20.2%	0.0%	0.0%		
Rotating shift						3.739 (4)	0.442
No	26.9%	18.7%	16.1%	0.0%	0.0%		
Rotated shift	73.1%	81.3%	83.9%	100.0%	100.0%		
How often do you have to work back-to-back shifts, for example, working two 12-h shifts in a row?						13.153 (16)	0.662
Never	3.8%	6.6%	4.0%	0.0%	0.0%		
Occasionally	46.2%	31.9%	42.7%	50.0%	0.0%		
Sometimes	19.2%	36.3%	30.6%	12.5%	100.0%		
Often	7.7%	13.2%	12.9%	12.5%	0.0%		
Always	23.1%	12.0%	9.7%	25.0%	0.0%		
How many overtime hours do you work per week (on average)						163.711 (16)	0.001 *
None	0.0%	6.6%	3.2%	0.0%	0.0%		
12 h	30.8%	54.9%	70.2%	62.5%	0.0%		
24 h	19.2%	14.3%	9.7%	0.0%	0.0%		
3 h	7.7%	0.0%	0.0%	0.0%	0.0%		
48 h	3.8%	3.3%	0.8%	0.0%	0.0%		
8 h	0.0%	0.0%	0.8%	0.0%	100.0%		
Others	38.5%	20.9%	15.3%	37.5%	0.0%		
How often are you required to work overtime						11.146 (12)	0.516
Daily	0.0%	1.1%	0.0%	0.0%	0.0%		
Weekly	30.8%	22.0%	16.9%	25.0%	100.0%		
Occasionally	69.2%	71.4%	79.0%	62.5%	0.0%		
Never	0.0%	5.5%	4.0%	12.5%	0.0%		
Are you satisfied with your current shift schedule						3.202 (4)	0.525
Yes	26.9%	33.0%	39.5%	50.0%	0.0%		
No	73.1%	67.0%	60.5%	50.0%	100.0%		
What is the current nurse-to-patient ratio in your ICU						36.170 (20)	0.015 *
1:1	0.0%	14.3%	17.7%	50.0%			
1:2	57.7%	54.9%	48.4%	37.5%	100.0%		
1:3	34.6%	19.8%	14.5%	0.0%	0.0%		
1:4	3.8%			0.0%	0.0%		
Others	3.8%	11.0%	19.3%	12.5%	0.0%		
How often does the nurse-to-patient ratio exceed the standard recommendation in your ICU						22.381 (12)	0.033 *
Rarely	15.4%	13.2%	10.5%	50.0%	100.0%		
Occasionally	65.4%	75.8%	80.6%	37.5%	0.0%		
Frequently	15.4%	8.8%	8.9%	12.5%	0.0%		
Always	3.8%	2.2%	0.0%	0.0%	0.0%		
How does the nurse patient ratio affect patient outcomes						31.382 (8)	0.001 *
Negatively	0.0%	9.9%	10.5%	25.0%	0.0%		
Neutral	42.3%	71.4%	75.0%	50.0%	0.0%		
Positively	57.7%	18.7%	14.5%	25.0%	100.0%		
How often do you feel that the staffing levels are adequate to meet the patient’s needs						20.175 (16)	0.190
Never	3.8%	4.3%	4.8%	0.0%	0.0%		
Rarely	19.2%	44.0%	38.7%	62.5%	100.0%		
Sometimes	61.5%	44.0%	53.2%	37.5%	0.0%		
Often	11.5%	7.7%	3.2%	0.0%	0.0%		
Always	4.0%	0.0%	0.0%	0.0%	0.0%		
What challenges do you face due to the current nurse–patient ratio						17.530 (12)	0.131
Increased workload							
No	26.9%	49.5%	40.3%	12.5%	0.0%		
Increased workload	73.1%	50.5%	59.7%	87.5%	100.0%		
Reduced time for patient care						18.572 (8)	0.017 *
No	30.8%	49.5%	40.5%	12.5%	0.0%		
Reduced time for patient care	69.2%	50.5%	59.5%	87.5%	100.0%		
Increased stress						9.554 (4)	0.049 *
No	61.5%	41.8%	34.7%	62.5%	100.0%		
Increased stress	38.5%	58.2%	65.3%	37.5%	0.0%		
Higher risk of errors						7.128 (4)	0.129
No	73.1%	52.7%	61.3%	87.5%	100.0%		
Higher risk of errors	26.9%	47.3%	38.7%	12.5%	0.0%		
Others						1.065 (4)	0.900
No	96.2%	94.5%	95.2%	87.5%	100.0%		
Other	3.8%	5.5%	4.8%	12.5%	0.0%		

Notes: * significance at 0.01 confidence interval.

**Table 6:** Associations with workload and staffing and detachment among ICU nurses.

Variables	How Often Do You Feel Detached or Indifferent Towards Your Patients?					Chi-Square (df)	*p*-Value
	Never	Rarely	Sometimes	Often	Always		
How many hours do you work per week						42.280 (16)	0.001 *
12	0.0%	1.4%	0.8%	0.0%	0.0%		
46	0.0%	0.0%	1.6%	2.6%	16.7%		
48	60.0%	93.2%	89.8%	79.5%	83.3%		
60	20.0%	5.4%	7.8%	15.4%	0.0%		
72	20.0%	0.0%	0.0%	2.5%	0.0%		
What is the average length (hours) per day shift						2.434 (4)	0.656
12	100.0%	100%	100.0%	100.0%	100.0%		
What types of shifts do you work							
Day shift						7.559 (8)	0.478
No	100.0%	76.7%	84.3%	87.2%	100.0%		
Day shift		23.3%	15.7%	12.8%	0.0%		
Night shift						9.971 (8)	0.267
No	60.0%	72.6%	84.3%	84.6%	100.0%		
Night shift	40.0%	27.4%	15.7%	15.4%	0.0%		
Rotating shift						6.679 (4)	0.154
No	40.0%	24.7%	14.2%	15.4%	0.0%		
Rotated shift	60.0%	75.3%	85.8%	84.6%	100.0%		
How often do you have to work back-to-back shifts, for example, working two 12-h shifts in a row?						38.594 (16)	0.001 *
Never	0.0%	8.2%	4.7%	0.0%	0.0%		
Occasionally	20.0%	35.6%	44.1%	35.9%	16.7%		
Sometimes	40.0%	37.0%	32.3%	17.9%	16.7%		
Often		11.0%	11.8%	12.8%	50.0%		
Always	40.0%	8.2%	7.1%	33.4%	16.6%		
How many overtime hours do you work per week (on average)						62.178 (24)	0.001 *
None	20.0%	6.8%	1.6%	5.1%	0.0%		
12 h	20.0%	54.9%	63.7%	66.7%	33.3%		
24 h	40.0%	16.4%	12.6%	0.0%	0.0%		
3 h	0.0%	0.0%	0.0%	5.1%	0.0%		
48 h	0.0%	5.5%	0.8%	0.0%	0.0%		
8 h	0.0%	0.0%	0.0%	2.6%	16.7%		
Others	20.0%	16.4%	21.3%	20.5%	50.0%		
How often are you required to work overtime						13.700 (12)	0.320
Daily	0.0%	1.4%	0.0%	0.0%	0.0%		
Weekly	40.0%	27.3%	17.3%	17.9%	16.7%		
Occasionally	60.0%	69.9%	74.8%	82.1%	83.3%		
Never	0.0%	1.4%	7.9%	0.0%	0.0%		
Are you satisfied with your current shift schedule						8.890 (4)	0.064
Yes	40.0%	39.7%	28.3%	48.7%	66.7%		
No	60.0%	60.3%	71.7%	51.3%	33.3%		
What is the current nurse to patient ratio in your ICU						42.941 (20)	0.002 *
1:1	0.0%	6.8%	17.3%	28.2%	16.7%		
1:2	100.0%	52.2%	54.3%	33.3%	83.3%		
1:3	0.0%	34.2%	13.2%	7.7%	0.0%		
1:4	0.0%	0.0%	0.8%	0.0%	0.0%		
Others	0.0%	6.8%	14.1%	30.8%	0.0%		
How often does the nurse-to-patient ratio exceed the standard recommendation in your ICU						15.563 (12)	0.212
Rarely	20.0%	8.2%	12.6%	20.5%	50.0%		
Occasionally	60.0%	76.8%	79.5%	66.7%	50.0%		
Frequently	20.0%	12.3%	7.1%	12.8%	0.0%		
Always	0.0%	2.7%	0.8%	0.0%	0.0%		
How does the nurse–patient ratio affect patient outcomes						10.314 (8)	0.244
Negatively	20.0%	6.8%	8.7%	12.8%	33.3%		
Neutral	60.0%	63.1%	74.0%	69.3%	50.0%		
Positively	20.0%	30.1%	17.3%	17.9%	16.7%		
How often do you feel that the staffing levels are adequate to meet the patient’s needs						38.575 (16)	0.001 *
Never	20.0%	5.5%	3.1%	0.0%	33.3%		
Rarely	40.0%	42.5%	38.6%	41.0%	16.7%		
Sometimes	0.0%	42.5%	53.5%	59.0%	50.0%		
Often	40.0%	9.5%	3.9%	0.0%	0.0%		
Always	0.0%	0.0%	0.9%	0.0%	0.0%		
What challenges do you face due to the current nurse–patient ratio							
Increased workload						65.538 (12)	0.001 *
No	40.0%	58.9%	31.5%	41.0%	33.3%		
Increased workload	60.0%	41.1%	68.5%	59.0%	66.7%		
Reduced time for patient care						29.080 (8)	0.001 *
No	40.0%	47.9%	39.4%	56.4%	66.7%		
Reduced time for patient care	60.0%	52.1%	60.6%	43.6%	33.3%		
Increased stress						14.730 (4)	0.005 *
No	80.0%	54.8%	30.7%	43.6%	50.0%		
Increased stress	20.0%	45.2%	69.3%	56.4%	50.0%		
Higher risk of errors						0.357 (4)	0.986
No	60.0%	61.6%	59.8%	61.5%	50.0%		
Higher risk of errors	40.0%	38.4%	40.2%	38.5%	50.0%		
Others						12.332 (4)	0.015 *
No	100.0%	95.9%	97.6%	84.6%	83.3%		
Other		4.1%	2.4%	15.4%	16.7%		

Notes: * significance at 0.01 confidence interval.

**Table 7:** Self-reported measures to reduce stigma around seeking help for burnout.

What Measures Do You Think Could Be Implemented to Reduce Stigma Around Seeking Help for Burnout	Frequency	Percentage
Education and training in mental health		
No	133	53.2
Yes	117	46.8
Anonymous support programmes		
No	97	38.8
Yes	153	61.2
Regular mental health check-ins		
No	123	49.2
Yes	127	50.8
Encouragement from the leader’s hip to seek help		
No	77	30.8
Yes	173	69.2
Others		
No	237	94.8
Yes	13	5.2

## Data Availability

Data used for the paper are available in a de-identified format from the corresponding author upon reasonable request.

## Supplementary Materials

The following supporting information can be downloaded at: https://www.mdpi.com/article/10.3390/ijerph23060757/s1, File S1: Questionnaire.

## Abbreviations

The following abbreviations are used in this manuscript:

ICU	Intensive Care Unit
SCFHS	Saudi Commission for Health Science
SPSS	Statistical Package for Social Science
