# Supplementary material for: Prevalence of Burnout and Associated Work-Related Factors Among Intensive Care Unit Nurses at Tertiary Healthcare Setting, Riyadh, Saudi Arabia
Source: Int J Environ Res Public Health. 2026 Jun 4;23(6):757. doi: 10.3390/ijerph23060757 (PMC13299354; doi:10.3390/ijerph23060757)
Supplement: Supplementary file 1 [file ijerph-23-00757-s001.zip › ijerph-4228079-supplementary.pdf]

Supplementary File 1 (S1)

Questionnaire

STUDY TOPIC: PREVALENCE OF BURNOUT AND ASSOCIATED FACTORS AMONGST NURSES WORKING IN AN INTENSIVE CARE UNIT AT KING FAHAD MEDICAL CITY HOSPITAL, SAUDI ARABIA

Please fill in the required questions from question 1 to 43. When you encounter a question that includes the option " please specify, or other, please kindly follow these guidelines. In the space provided, kindly provide additional details to explain your choice where indicated. This will help us better understand your perspective

SOCIO-DEMOGRAPHIC INFORMATION

1. Age

   ____

2. Gender
   - Male
   - Female
   - Other: ____________________

3. Marital status
   - Married
   - Single
   - Widowed
   - Divorced
   - Other: ____________________

4. Who do you currently live with / If any?
   - My children
   - My family
   - Alone
   - Other: ____________________

5. How often do you see your family (if not living with them)/ if any?
   - Every 3 months
   - Every 6 months
   - Annually
   - Other: ____________________

6. How would you describe your current support system?
   - Strong
   - Moderate
   - Weak

7. What is your highest educational level?
- Doctorate
- Master's degree
- Bachelor’s degree
- Diploma

8. Do you have any ICU qualification?
- Yes
- No

WORK EXPERIENCE

9. How many years of experience do you have as a nurse?
- Less than 1 year
- 1 to 3 years
- 4 to 5 years
- 6 to 10 years
- 11 to 15 years
- 16 to 20 years
- 21 years and above

10. Years of experience in the Intensive Care Unit?
- Less than 1 year
- 1 to 3 years
- 4 to 5 years
- 6 to 10 years
- 11 to 15 years
- 16 to 20 years
- 21 years and above

11. What is your current role?
- Staff Nurse
- Charge Nurse

12. What type of ICU do you work in?
- NICU
- PCVICU
- PICU
- CCU
- NCCU
- GICU
- ICU D
- ACVICU
- ED Critical Care

OCCUPATIONAL RELATED FACTORS

13. How many hours do you work per week?

____

14. What is the average length per day shift?

____

15. What types of shifts do you work?
(*click all that apply*)
- Day shift
- Night shift
- Rotating shifts

16. How often do you have to work back-to-back shifts, for example, working two 12-hour shifts in a row?
- Never
- Occasionally
- Sometimes
- Often
- Always

17. How many overtime hours do you work per week (on average)?

____

18. How often are you required to work overtime?
- Daily
- Weekly
- Occasionally
- Never

19. Are you satisfied with your current shift schedule?
- Yes
- No

20. Any additional comments on how shifts affect your well-being?
____
____

21. What is the current nurse to patient ratio in your ICU
- 1:1
- 1:2
- 1:3
- 1:4
- Other: ____________________

22. How often does the nurse-to-patient ratio exceed the standard recommendation in your ICU?
- Rarely
- Occasionally

- Frequently
- Always

23. How does the nurse patient ratio affect patient outcomes?
- Positively
- Neutral
- Negative

24. In your own opinion, how often do you feel that the staffing levels are adequate to meet the patient's needs?
- Always
- Often
- Sometimes
- Rarely
- Never

25. What challenges do you face due to the current nurse patient ratio?
(*click all that apply*)
- Increased workload
- Reduced time for patient care
- Increased stress
- Higher risk of errors
- Other: ____________________

BURNOUT ASSESSMENT

26. How often do you take breaks during your shift?
- Regularly
- Occasionally
- Rarely
- Never

27. How often do you feel emotionally drained after your shift?
- Always
- Often
- Sometimes
- Rarely
- Never

28. How often do you feel detached or indifferent towards your patients?
- Always
- Often
- Sometimes
- Rarely

- Never

29. Do you experience physical symptoms (e.g., headaches, fatigue) related to work stress?
    - Always
    - Often
    - Sometimes
    - Rarely
    - Never

30. Do you feel supported by colleagues during stressful situations?
    - Always
    - Often
    - Sometimes
    - Rarely
    - Never

31. Have you ever sought support (e.g., counseling or peer support) for stress or trauma?
    - Yes
    - No

ACCESS TO RESOURCES

32. How would you rate your resources in your unit, for example, equipment, supplies, and professional development?
    - Excellent
    - Good
    - Fair
    - Poor

33. Do you think that insufficiency of resources contributes to your stress?
    - Strongly agree
    - Agree
    - neutral
    - Disagree
    - Strongly disagree.

34. How often do you encounter situations where resources are inadequate during your shifts?
    - Always
    - Often
    - Sometimes
    - Rarely
    - Never

UNIT MEETINGS

35. How often do unit meetings occur in your department?

- Weekly
- Monthly
- Rarely
- Never

36. Do you find unit meetings helpful for addressing concerns and improving work conditions?
    - Very helpful
    - Somewhat helpful
    - Neutral
    - Somewhat unhelpful
    - Very unhelpful

37. How often do you feel comfortable voicing concerns during unit meetings?
    - Always
    - Often
    - Sometimes
    - Rarely
    - Never

JOB SATISFACTION

38. How satisfied are you with your current job in ICU?
    - Very satisfied
    - Satisfied
    - Neutral
    - Dissatisfied
    - Very dissatisfied

39. How would you rate work-life balance?
    - Excellent
    - Good
    - Fair
    - Poor

40. Do you feel fatigued when you get up in the morning and you must go to work?
    - Always
    - Often
    - Sometimes
    - Rarely
    - Never

41. Do you feel a sense of personal accomplishment from your work?
    - Always
    - Often
    - Sometimes

- Rarely
- Never

42. Do you feel that you are making an impact on patients' lives?
    - Always
    - Often
    - Sometimes
    - Rarely
    - Never

SUGGESTIONS

43. What measures do you think could be implemented to reduce stigma around seeking help for burnout? (Select all that apply)
    - Education and training in mental health
    - Anonymous support programs
    - Regular mental health check-ins
    - Encouragement from the leader's hip to seek help
    - Other: ____
